# A rabbit model for outer retinal atrophy caused by surgical RPE removal

**DOI:** 10.1007/s00417-023-06014-3

**Published:** 2023-03-28

**Authors:** Sami Al-Nawaiseh, Christina Krötz, Annekatrin Rickmann, Claudine Strack, Anja Germann, Hagen von Briesen, Peter Szurman, André Schulz, Boris V. Stanzel

**Affiliations:** 1 Eye Clinic Sulzbach, Knappschaft Hospital Saar, Sulzbach/Saar, Germany; 2 Fraunhofer Institute for Biomedical Engineering, Sulzbach/Saar, Germany; 3 Department of Ophthalmology, University of Bonn, Bonn, Germany; 4 Klaus Heimann Eye Research Institute (KHERI), Sulzbach/Saar, Germany

**Keywords:** Mechanical debridement, Subretinal surgery, Animal model, RPE degeneration, Rabbit, Cell therapy

## Abstract

**Purpose:**

We aimed to establish a rabbit model with retinal atrophy induced by an iatrogenic retinal pigment epithelium (RPE) removal, for future testing of the efficacy and safety of cell therapy strategies.

**Methods:**

A localized detachment of the retina from the RPE/choroid layer was created in 18 pigmented rabbits. The RPE was removed by scraping with a custom-made extendable loop instrument. The resulting RPE wound was observed over a time course of 12 weeks with optical coherence tomography and angiography. After 4 days (group 1) and 12 weeks (group 2), histology was done and staining with hematoxylin and eosin, as well as immunofluorescence performed to further investigate the effects of debridement on the RPE and the overlying retina.

**Results:**

Already after 4 days, we observed a closure of the RPE wound by proliferating RPE and microglia/macrophage cells forming a multilayered clump. This pattern continued over the observation time course of 12 weeks, whereby the inner and outer nuclear layer of the retina became atrophic. No neovascularization was observed in the angiograms or histology. The observed changes were limited to the site of the former RPE wound.

**Conclusions:**

Localized surgical RPE removal induced an adjacent progressive retinal atrophy. Altering the natural course of this model may serve as a basis to test RPE cell therapeutics.

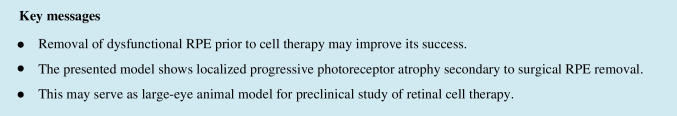

**Supplementary Information:**

The online version contains supplementary material available at 10.1007/s00417-023-06014-3.

## Introduction

The majority of age-related macular degeneration (AMD) patients have no effective treatment option available. While neovascular forms of AMD can now be effectively treated by repetitive injections of vascular endothelial growth factor (VEGF) inhibitors for preventing further loss of vision, no therapy is available for the dry late manifestation (geographic atrophy) [1]. A common denominator of the disease seems the impairment of the retinal pigment epithelium (RPE), a cell monolayer located between the choroid and photoreceptors and superimposed on a thin, acellular, five-layered extra cellular matrix called Bruch’s membrane (BM) [2]. The replacement of these dysfunctional or lost submacular RPE cells therefore represents a meaningful therapeutic goal [3–5].

Various clinical treatment concepts with RPE replacement have been tried so far, such as macular translocation, peripheral RPE cell suspensions, and autologous RPE/choroidal grafts [3, 4, 6, 7]. However, either no or only limited visual acuity improvements have been achieved [4].

Recently, novel methods to transplant pluripotent stem cell-derived RPE cells (PSC-RPE) have become available, either as a cell suspension or as a bioengineered monolayer [2]. In this context, it is worth noting that a US FDA Phase 1/2 clinical trial on transplantation of human embryonic stem cell-derived RPE cells (hES-RPE) suspensions into patients with advanced dry AMD has been completed at several US centers [8, 9]. Other clinical studies were conducted showing the survival and integration of PSC-RPE delivered under the retina on a patch [10–13].

So far, ongoing clinical studies with stem cell-derived RPE replacement in atrophic AMD have conducted the transplantation on to an intact, but presumably less functional, RPE in the so-called transition zone. From many points of view, it is desirable to perform a complete replacement of the degenerated RPE with a healthy RPE graft. In a mouse RPE toxicity model for example, it was shown that sodium iodate-necrosed host RPE cells could be completely replaced as a single layer by xenotransplantation of a hES-RPE suspension [14]. Since it is highly unlikely that sodium iodate or other RPE toxic substances would be used to remove the dysfunctional RPE, local measures are needed to remove the RPE in the graft target area. For this purpose, we have developed a simple instrument that has been successfully tested in three species (rabbit, pig, and monkey) [15]. Although our instrument has proven to be effective in short-term experiments [16], a subretinal injection of viscoelastics was needed to eliminate its flute needle effect.

The aim of this animal experiment series was to characterize a rabbit model with circumscribed retinal and choroidal atrophy surrounding the area of the created RPE wound using an improved instrument to enable testing of RPE cell therapeutics in future.

## Materials and methods

### Animals

We purchased 18 female and male Dutch Belted rabbits weighing 2 to 2.5 kg (Covance Research Products, Denver/PA, USA). All procedures were approved by the state regulatory authorities of Belgium study code 18–080 and were in accordance with the Association for Research in Vision and Ophthalmology Statement for the Use of Animals in Ophthalmic and Vision Research. Animals were held indoors in a specialized facility in an air-conditioned room with temperatures between 18 °C and 20 °C, exposure to 12 h of daylight, in standardized individual cages with free access to food and water.

### Study design

Animals were divided into 2 groups, depending on follow-up time (Table 1). In 15 rabbits, native RPE was removed by scraping, whereas in 3 animals the RPE was left untouched serving as a control. After surgery, follow-up examinations were performed after 4 days, 1, 2, 4, and 12 weeks post-operatively using scanning laser ophthalmoscopy and spectral domains of optical coherence tomography (SLO/SD-OCT), fluorescein angiography (FA), and indocyanine green angiography (ICGA) (Spectralis®, Heidelberg Engineering, Germany). For histological examination, 8 animals were euthanized after their last examination on day 4 and the remaining 10 animals were euthanized 12 weeks after surgery.

**Table 1:** Overview on animals and surgical procedures

Rabbit Nr	Gender	Operated eye	Procedures	Histological processing after operation
1–4	Female	Left	Two retinotomies followed by RPE scraping	4 days
5–8	Male	Left	Two retinotomies followed by RPE scraping	4 days
9–11	Female	Left	Two retinotomies followed by RPE scraping	84 days
12–15	Male	Left	Two retinotomies followed by RPE scraping	84 days
16–17	Female	Left	Two retinotomies without RPE scraping (control)	84 days
18	Male	Left	Two retinotomies without RPE scraping (control)	84 days

### Surgery

**Fig. 1:**
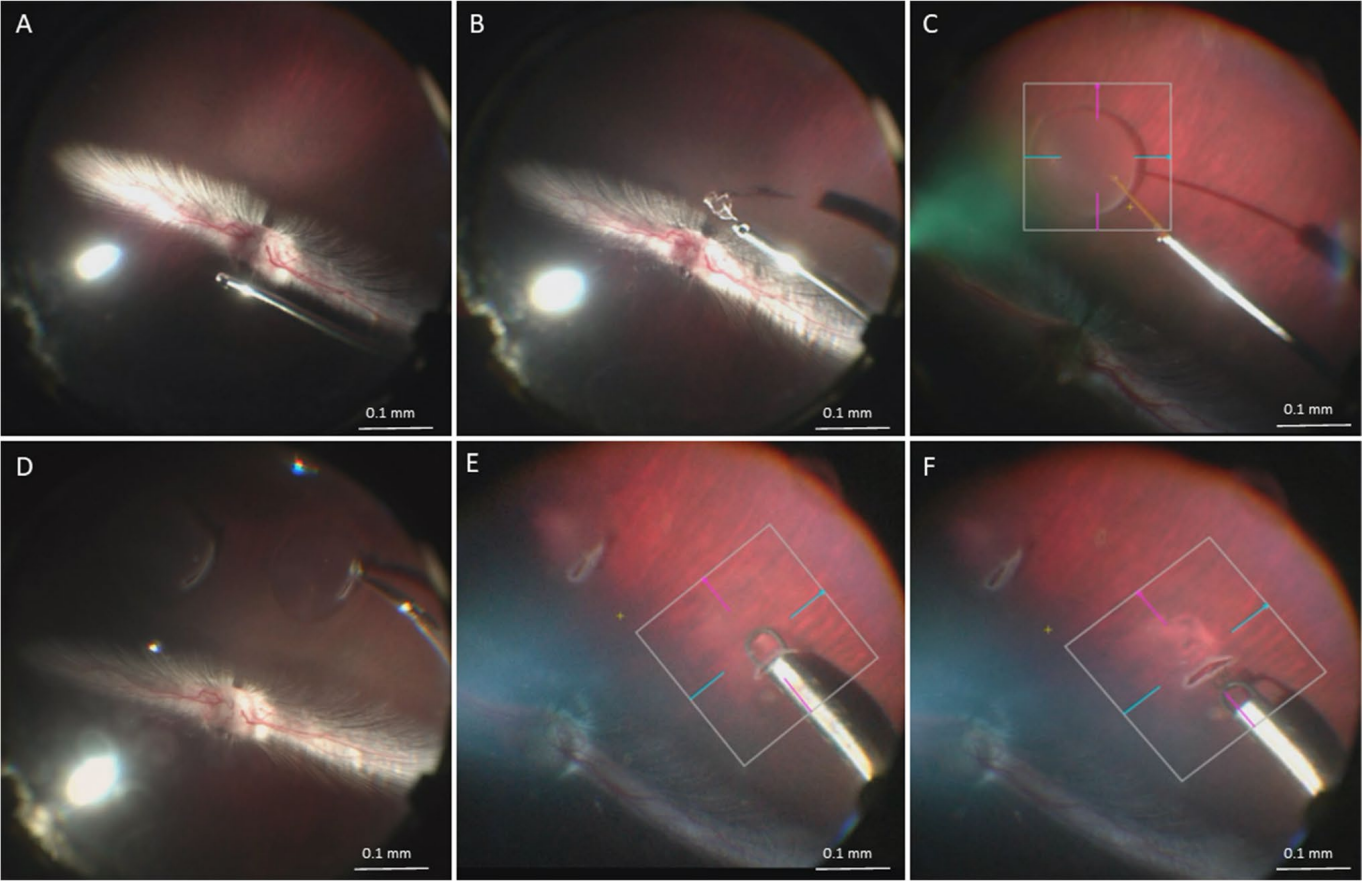
Intraoperative imaging of key steps in surgical procedure. (**A**) 25G vitrectomy over the optic nerve to disrupt the Alae canalis Cloqueti [39]. (**B**) Triamcinolone-assisted posterior vitreous detachment. (**C**) Formation of circumscribed bleb retinal detachment. (**D**) Performing the retinotomy. (**E**) Bleb during subretinal scraping. (**F**) Bleb after scraping, note the removed RPE still sticking to scraping instrument at tip of pink miOCT scan line

As shown in Fig. 1 and Vid. S1, 18 Dutch Belted rabbits (2–2.5 kg) underwent surgery after intravenous induction of general anesthesia with ketamine (25 mg/kg) and xylazine (2 mg/kg). Triamcinolone-assisted, 25 gauge (G) 3 port vitrectomy was performed in the left eye, including induction of a posterior vitreous detachment. Subsequently, 1–2 localized circumscribed, bleb-shaped retinal detachments (bRD) were created by manual subretinal injection of 20–30 μL balanced salt solution (BSS) via a 25/38 G subretinal cannula (MedOne Surgical Inc., Sarasota/ FL, USA) connected to a 100 μL syringe (Hamilton Germany GmbH, Gräfelfing, Germany). A roughly 1.5 mm wide retinotomy was made using 23 G vertical retinal scissors (Geuder, Heidelberg, Germany) after which a RPE wound was created by scraping with a custom sling instrument made from prolene suture material (Geuder, Heidelberg, Germany) (Vid. S2) [16]. An intraocular tamponade was not required.

### Post-operative SLO/SD-OCT imaging

The rabbit was anesthetized, and pupils dilated as above. Application of a corneal lubricant (Optive®, Allergan, Frankfurt, Germany) was done at least every 5 min to moisturize the eye and maintain clear SD-OCT imaging; then, we attached a steel platform to the headrest to stabilize the animal in the required position. We used a 30-degree lens and the following parameters for optimal optical coherence tomography (OCT) imaging: 30 degrees settings for single line scans with automated real-time tracking (ART) mode set to 100 (averaging) and 20 × 20 degree settings for volume scans with ART mode set to 15; high resolution mode was not required. SLO infrared reflectance imaging aided us in finding the focal plane of the RPE-wound.

### Post-operative FA and ICGA

Following the SD-OCT, while still under anesthesia and similar positioning as described above, 0.2 mL sodium fluorescein solution (10%) (Alcon Pharma GmbH, Freiburg, Germany) and 0.5 mL containing 1.25 mg of indocyanine green (Diagnostic Green GmbH, Aschheim-Dornach, Germany) were injected in the marginal ear vein, and the images were captured immediately. Late-phase photos were acquired at every minute for a period of at least 15 min. Recordings of FA and ICGA were done separately to ensure optimal focus. We used a 30-degree lens, we acquired movies of early stages, followed by single images, while using the ART mean function for late/dark images. For ICGA, we reduced the laser intensity to 25–50% to acquire the early stages and returned it to 100% after ca. 2–3 min. After finishing the examination, we added dexamethasone 1 mg/g, neomycin sulfate 3,500 I.U./g, and polymyxin B sulfate 6,000 I.U./g ointment under the lid.

### Histologic sample collection

Following the last post-operative examination with SLO/SD-OCT at 4 days or 12 weeks after surgery (*N* = 8 at 4 days, 10 at 12 weeks), the animals were euthanized with 5 mL T61 (Intervet Deutschland GmbH, Unterschleißheim, Germany) in deep anesthesia as per above protocol. The heads were fixed via carotid-perfusion with 4% paraformaldehyde (PFA), buffered with 0.2 M phosphate buffered saline at pH 8.5. Eyes were enucleated and full thickness specimen (sclera, choroid, and retina) and then were prepared from the bleb areas with (*N* = 30) and without (*N* = 6) RPE manipulation; five RPE scraped regions were not sampled, as the scraping site was not well identifiable in hematoxylin and eosin (HE) staining and the histology did not match the corresponding OCT images. Two control regions from one right eye that did not undergo surgery were also prepared.

### Histological processing and photomicroscopy

The specimens were embedded in paraffin and cut into 5-µm thick sections. For morphological assessment, HE staining was performed. Pictures were taken on an Olympus IX71 microscope with an Olympus Dp72 camera.

Immunofluorescence staining was performed as follows: after dewaxing, the slides were placed in boiling citrate buffer, pH 6, and cooked for further 15 min in the microwave at 600 W to retrieve antigens. Before staining, the slides were allowed to cool down to room temperature for 20 min and then rinsed with tap water. Sections were transferred into a dark humid chamber, washed three times for 3 min each with Tris-buffered saline (TBS) with 0.1% Tween 20 (Sigma-Aldrich, Taufkirchen, Germany) (TBST) and then blocked with 5% Bovine Serum Albumin (BSA; VWR International GmbH, Darmstadt, Germany) in TBS for 45 min at room temperature. Primary antibodies and isolectin B4 were applied to the sections and incubated overnight at + 4 °C. The antibodies and isolectin B4 (IB4) were diluted in 1% BSA in TBS (Table 2). The next day, sections were brought to room temperature before continuing with washing as before and applying the secondary antibodies anti-mouse immunoglobulin G (IgG) Cy5 and anti-rabbit IgG Alexa647 (both Invitrogen, Carlsbad, CA, USA) 1:200 in 1% BSA in TBS for 1 h at room temperature in the dark humid chamber. Then, sections were washed three times for 3 min in TBST and nuclei were stained with 1 µg/mL 4′, 6-diamidino-2-phenylindole (DAPI; Invitrogen, Carlsbad, CA, USA) in TBS for 5 min. A last washing step for 5 min with TBS was done before sections were mounted with Shandon Immumount (Fisher Scientific, Schwerte, Germany). Fluorescence pictures were taken with a white light confocal laser scanning microscope (CLSM; TCS SP8, Leica Microsystems, Wetzlar, Germany). Microscope images were arranged with ImageJ 1.52p (National Institute of Health, USA) and overview images of the retina were generated with the Stitching plugin in ImageJ [17]. Staining controls are found in Fig. S1.

**Table 2:** List of markers used for immunohistochemistry

Marker	Concentration/dilution	Vendor	Cat. number	Description	Target
Ki67	1:200	Abcam, Berlin, Germany	Ab15580	Polyclonal antibody	Proliferating cells
Laminin	1:100	Invitrogen, Carlsbad, CA, USA	PA184171	Polyclonal antibody	Bruch’s membrane
Collagen IV	1:100	Abcam, Berlin, Germany	Ab1217147	Polyclonal antibody	Bruch’s membrane, vasculature
Pan cytokeratin	1:100	Bio-Techne, Wiesbaden, Germany	NBP2-44,368–0.1 mg	Monoclonal antibody, Clone PAN-CK	Epithelial cells, RPE
ZO-1	1:100	Invitrogen, Carlsbad, CA, USA	339,100	Monoclonal antibody, Clone 1A12	Tight junctions
Isolectin B4 DyLight594	1:100	Vector Laboratories Inc., Burlingame, CA, USA	VEC-DL-1207	Lectin from the plant *Griffonia simplicifolia*. Binds to α-galactose residues on proteins and lipids	Endothelial cells, macrophages, and microglia
4′, 6-diamidino-2-phenylindole (DAPI)	1 µg/ml	Invitrogen, Carlsbad, CA, USA	10,184,322	Binds to AT rich regions of DNA	Cell nuclei

## Results

### Improvement of surgical RPE removal technique

We successfully operated all 18 animals and induced a total of 36 bRDs. The RPE underneath the bRD was removed by scraping in 30 cases with a revised loop instrument. The major improvement to our previously published loop instrument [16] is that no subretinal viscoelastic is required for RPE ablation. We were able to eliminate the instrument’s flute needle effect that causes bleb collapse due to passive fluid drainage via tip and handle of the instrument as a result of the intraoperative pressure gradient from inside versus outside the eye. With up to 3 brushes, it was possible to create a 5–9 mm^2^ RPE wound. In continuation of our previous work, we now investigated the effects of RPE removal up to 12 weeks.

### Short-term effects of RPE debridement

**Fig. 2:**
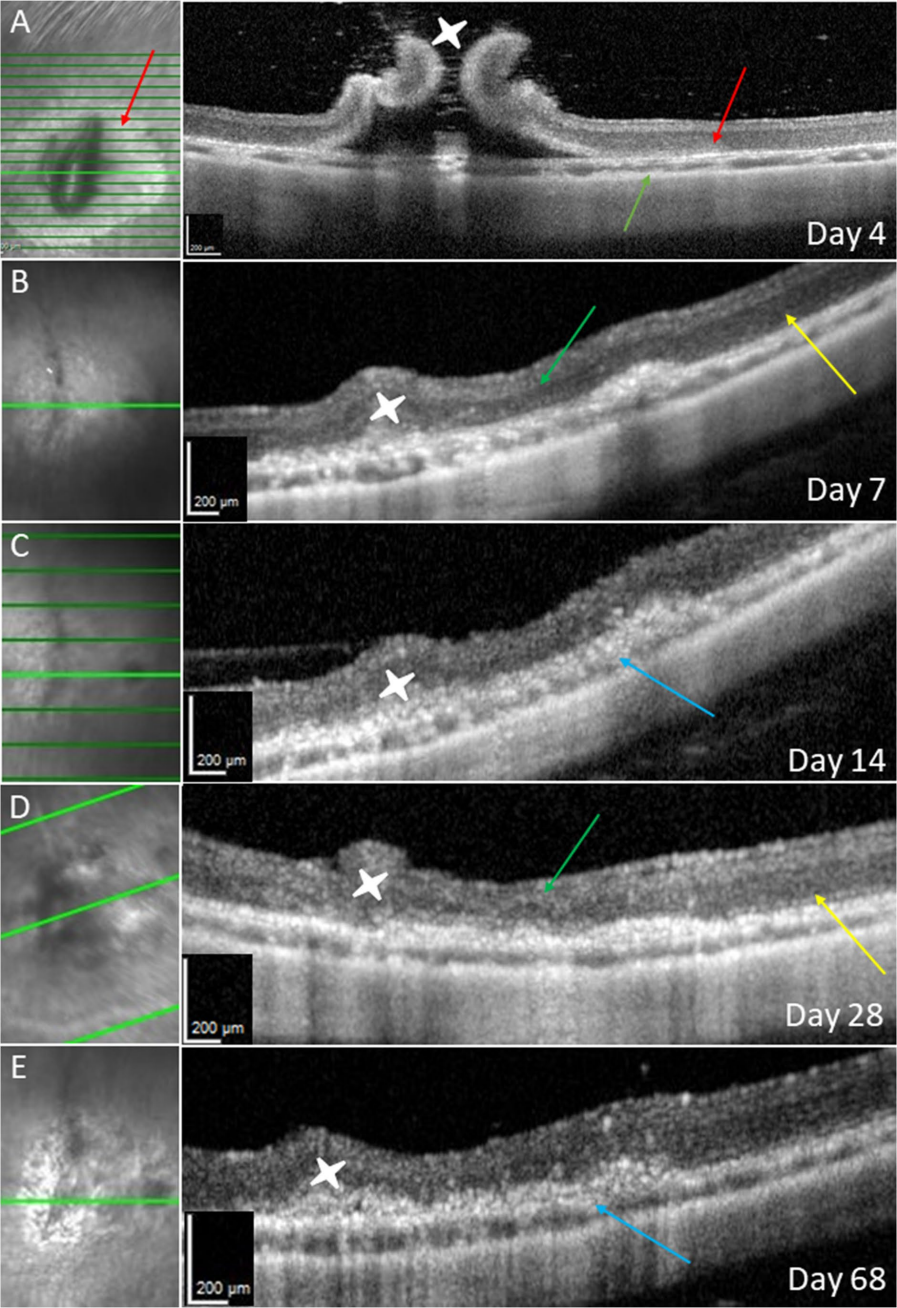
OCT after RPE scraping. Day 4 (**A**): bRD with RPE wound, red arrows show RPE defect and corresponding outer retinal change. The orange arrow shows the choroid with almost normal thickness, without iatrogenic bleeding. Follow-up on day 7 (**B**), 14 (**C**), 28 (**D**), and 68 (**E**): green arrows showing retinal atrophy above the RPE wound, and blue arrows showing RPE hypertrophy. White stars show the retinotomy with initially high standing retinal margins. The yellow arrows show the surrounding tissue without RPE manipulation

**Fig. 3:**
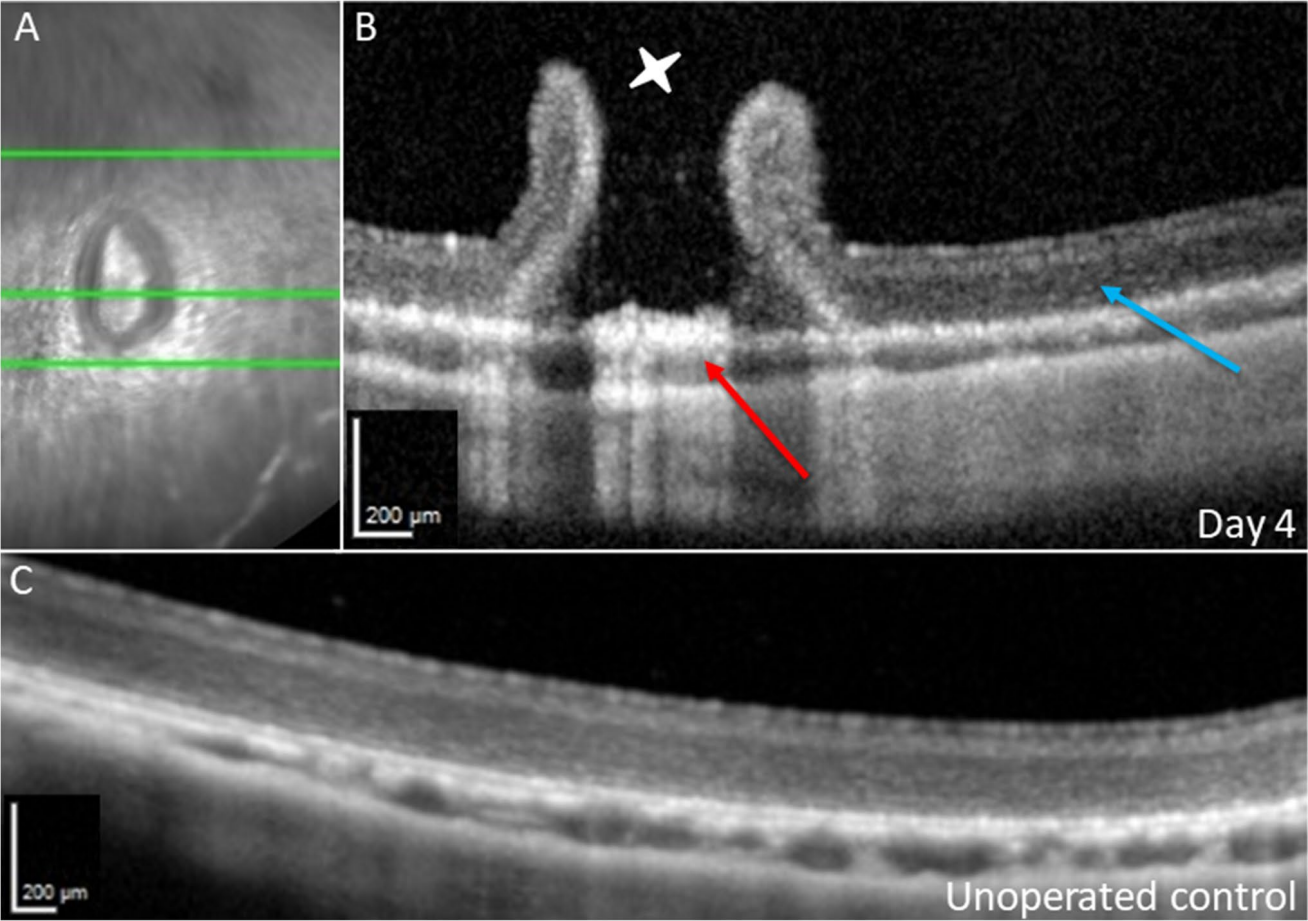
SD-OCT of bRD and retinotomy without RPE manipulation at 4 days post-operatively. (**A**) Infrared reflection image of the SLO. The green line marks the displayed cross-section in the corresponding SD-OCT image. (**B**) The red arrow shows central pigment epithelial hypertrophy at the retinotomy (white star) surrounded by raised retinal margins. Otherwise, near-normal retinal/choroidal OCT reflection bands can be discerned (blue arrow). In comparison, (**C**) shows a preoperative OCT, showing nearly the same reflection bands as in (**B**)

At follow-up on post-operative day 4, the optical media of the rabbits’ eyes were clear, with little post-operative inflammation. In the respective SD-OCT scans, the retina overlying the iatrogenic RPE wound showed an RPE irregularity and lacked intact ellipsoidal bands (Fig. 2A). By contrast, when the RPE was not scraped, an ellipsoid zone and RPE band remained distinguishable. An OCT of a normal rabbit retina before surgery is depicted in Fig. 3C and Fig. S2.

**Fig. 4:**
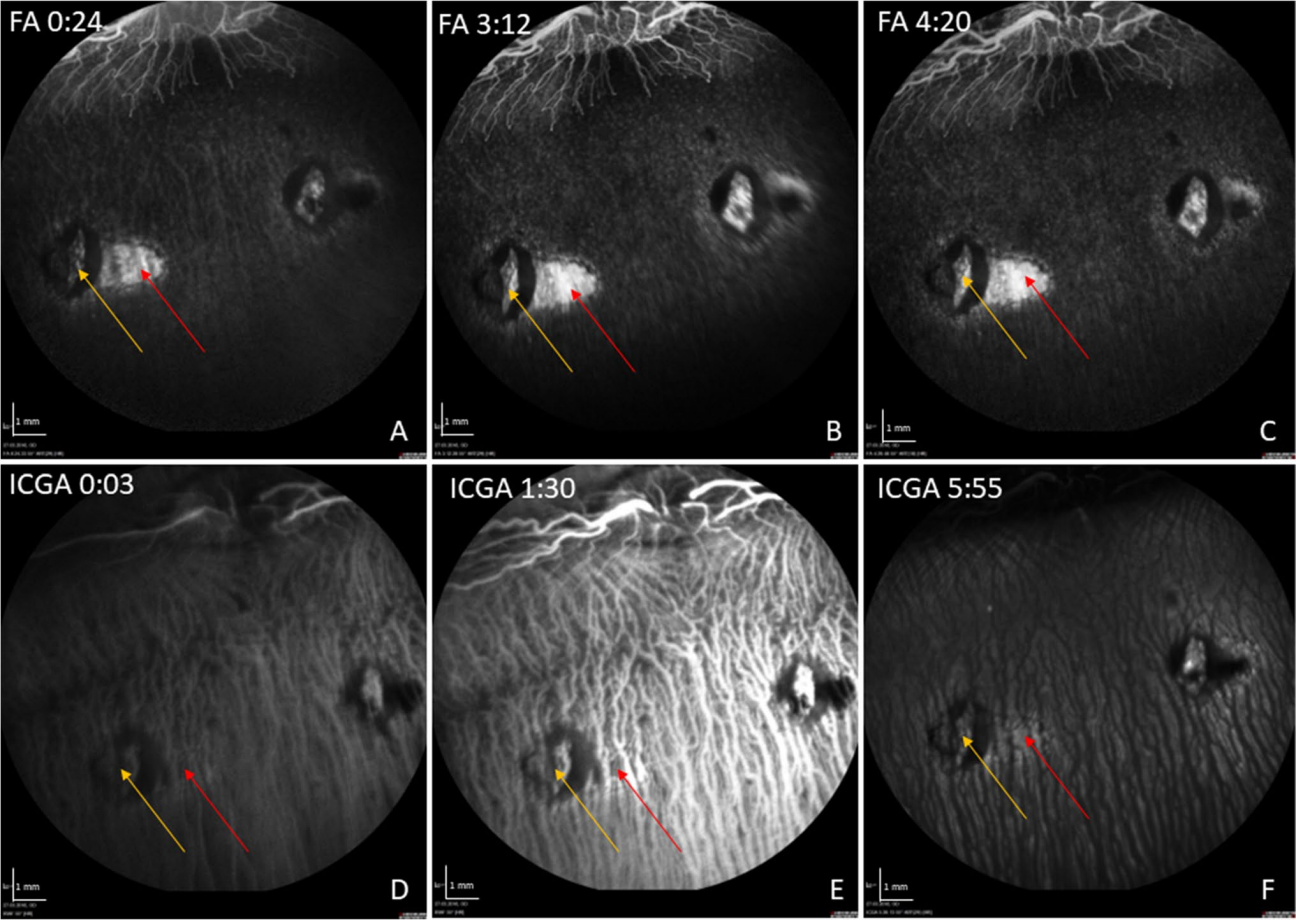
Representative FA/ICG with acquisition time (min:sec) at 1 week post-operatively. In the FA, a hyperfluorescence in the area of the induced RPE wound (red arrow) without leakage is shown over time (**A**–**C**). In the late phase of the ICGA, the RPE wounds appear hyperfluorescent with signal blockage corresponding to that seen in FA, suggesting it originates from hyperpigmentation of the formerly created RPE defect (**D**–**F**). The yellow arrow marks the retinotomy site

Angiographic examination of the retina showed a hyperfluorescence in FA and ICGA late phase at the site of the RPE wound (Fig. 4A–C, F), suggesting perfusion in the choriocapillaris. SD-OCT angiography obtained with the Spectralis 2 device (Heidelberg Engineering, Germany) failed due to motion artifacts (data not shown) and was therefore discontinued in subsequent follow ups. Choroidal thickness on SD-OCT was not altered in RPE wound areas versus unoperated regions. There was no sign of leakage in FA or branching vascular networks on ICGA, indicating that the surgical removal did not induce a choroidal neovascularization (CNV).

**Fig. 5:**
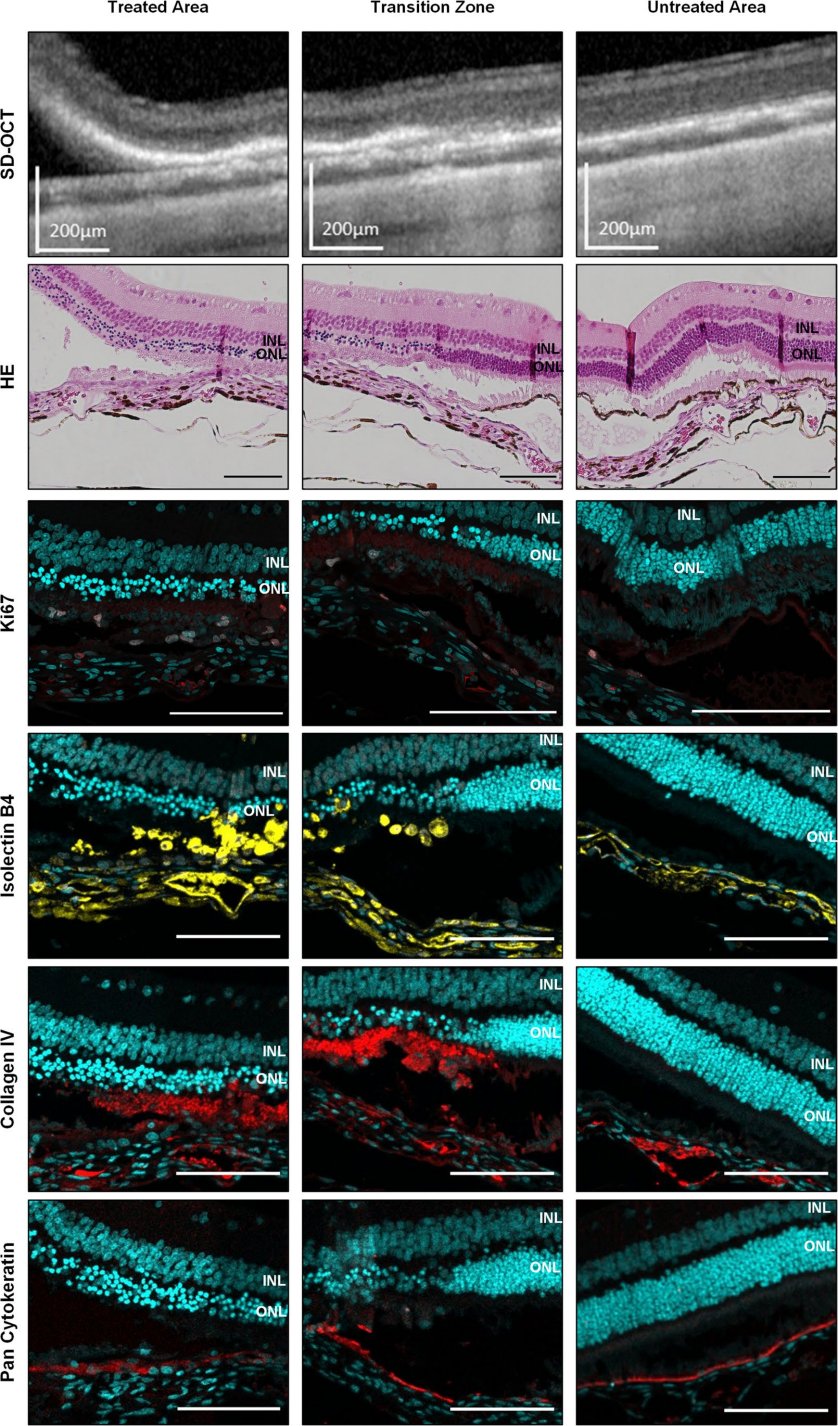
Visualization of the treated area, the transition zone, and untreated area as direct comparison by SD-OCT and histology at post-operative day 4. Hematoxylin and eosin (HE) staining already reveals atrophy of the photoreceptor outer segments and the outer nuclear layer (ONL) limited to the scraping site. The inner nuclear layer (INL) is not affected. Immunohistochemistry staining of the rabbit retina shows proliferating cells detected via Ki67 staining (red) which colocalize in part with subretinal microglia/macrophages shown by isolectin B4 staining (yellow). A strong deposition of collagen IV (red) is found around the scraping site beneath the ONL. Pan cytokeratin staining (red) for RPE already appears at the scraping site. Cell nuclei are stained with DAPI (cyan). Scale bar for SD-OCT = 200 µm, for histological images = 100 µm

Histological assessment of the specimens 4 days after surgery showed atrophic retinal regions above the areas where RPE was removed (Fig. 5). Especially the outer layers are affected which show photoreceptor degeneration and outer nuclear layer (ONL) thinning corresponding to the region of RPE removal. The limits of the RPE wound and where the healthy RPE begins could be nicely visualized by SD-OCT scan and the corresponding HE staining. Immunofluorescence staining for panCK showed already RPE cells that were repopulating the scraped site. These RPE cells exhibit only a weak ZO-1 staining, a marker for tight-junctions.

Normally, microglia reside in a quiescent state in the inner plexiform layer [18] (Fig. 5). Cell proliferation is seen at the RPE wound and to some extent at the margins of the retinotomy (Fig. 5 and Fig. S3). By using IB4 staining, we could see the presence of microglia and/or (monocyte derived) macrophages. Ki67 was used to assess cell proliferation and at the site of the RPE wound it colocalizes in part with microglia/macrophages. However, as no colocalization of panCytokeratin and Ki67 staining was shown, this suggests no involvement of RPE at the 4 days timepoint. The source of the collagen IV deposition is unclear, but colocalizes in some part with the proliferating microglia/macrophages (Fig. 5 and Fig. S3).

### Long-term effects of RPE debridement

In the following 12 weeks, we saw a complete closure of the retinotomy, increasing atrophy of the retina, and a hypertrophy of the RPE in OCT follow-up exams (Fig. 2B–E).

**Fig. 6:**
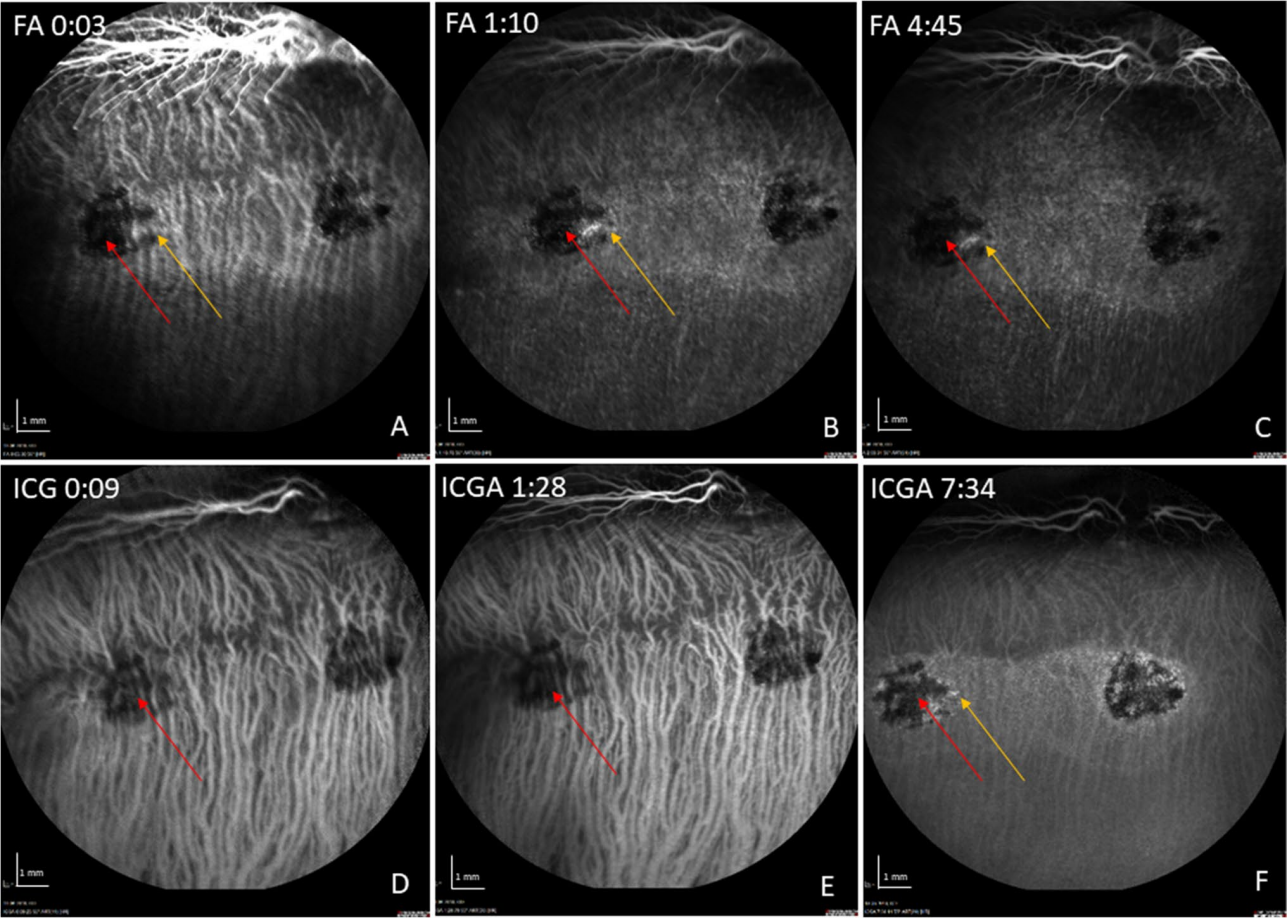
Representative FA/ICG with acquisition time (min:sec) at 12 weeks post-operatively. In the FA, a small staining in the area of the induced RPE wound appears at the edge of a hypofluorescent lesion (yellow arrow), likely resulting from fluorescein blockage due to RPE hyperpigmentation (red arrow) (**A**–**C**). In the ICG, choroidal perfusion remains intact under the blocked fluorescence of the pigmented lesion (**D**–**F**). The subtle ICG hyperfluorescence (yellow arrow) at the edges of the RPE lesion at mid late phase may suggest perfused choriocapillaris (**F**)

In the angiographic displays at 12 weeks after surgery, only subtle hyperfluorescence in the late phase of the FA could be shown in the scraping area (Fig. 6). The choriocapillaris could not be visualized satisfactorily over time due to blockage of the FA/ICG signal by accumulated pigment (Fig. 6).

**Fig. 7:**
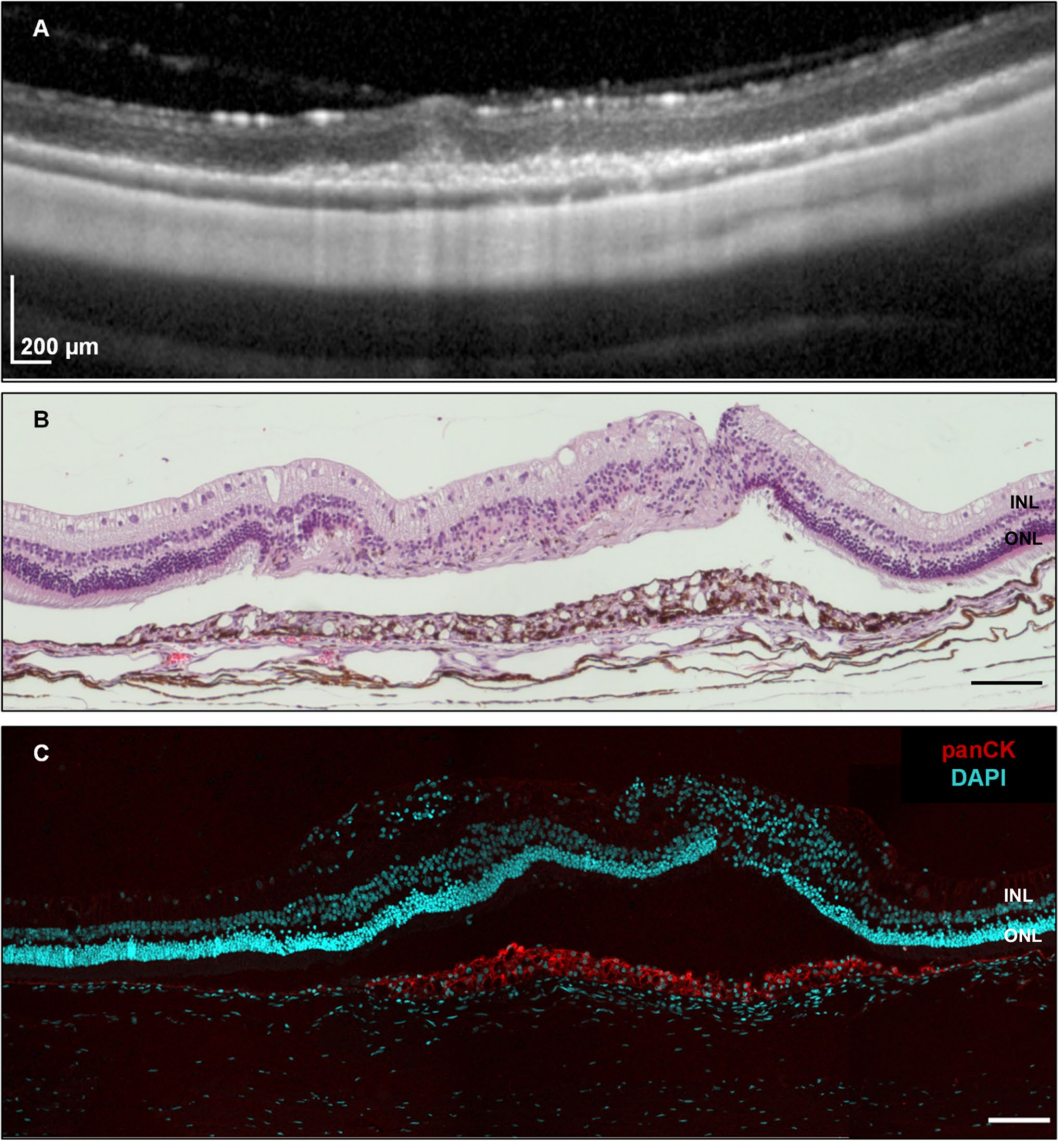
SD-OCT (**A**) and corresponding histology at 12 weeks post-operatively. HE staining (**B**) shows atrophic and disorganized outer and inner retinal layers overlying the region of the original RPE wound. RPE hypertrophy is visible in both the HE and immunofluorescence staining (**C**). Note the normal RPE morphology adjacent to the RPE wound. INL, inner nuclear layer; ONL, outer nuclear layer. Scale bar = 100 µm

**Fig. 8:**
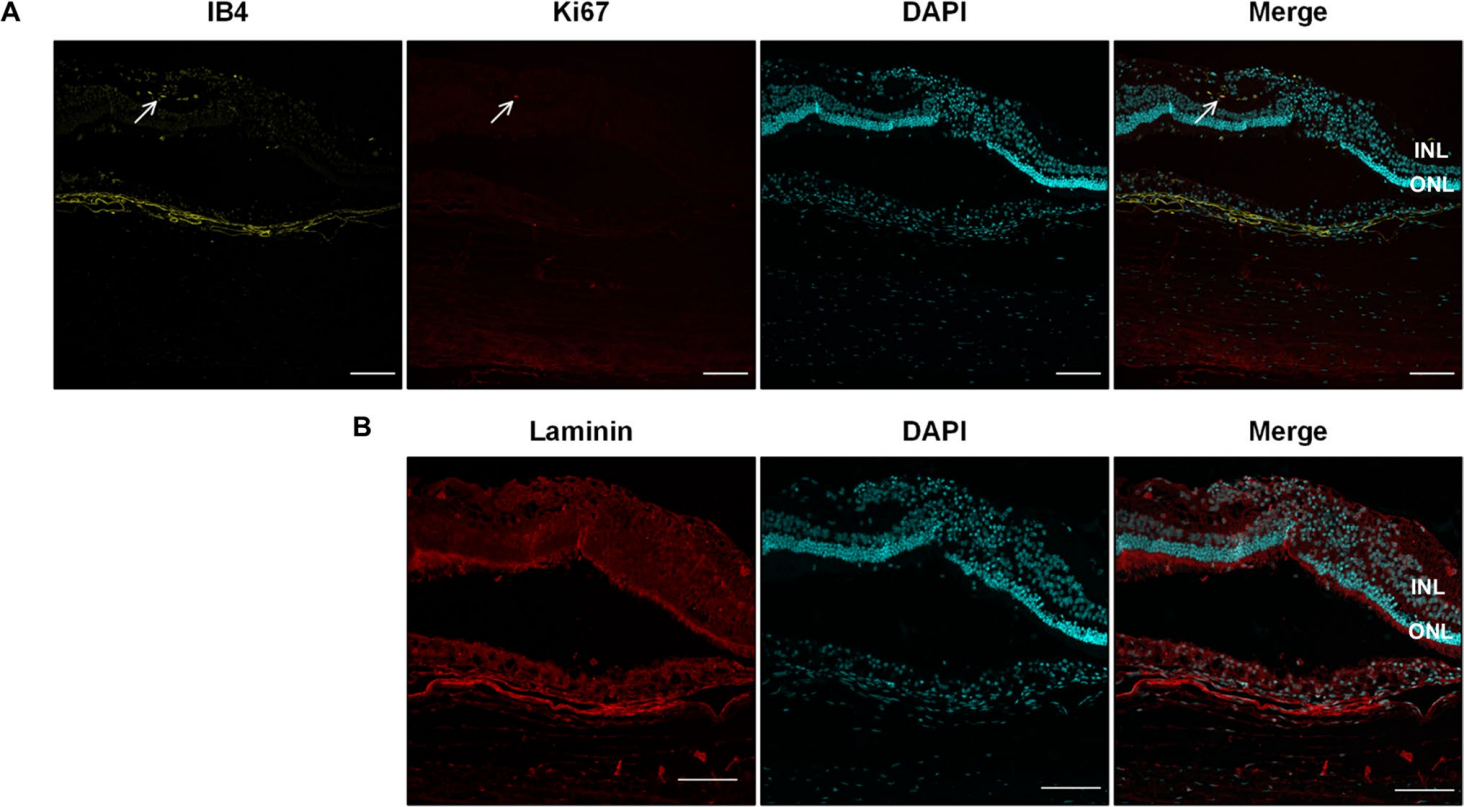
(**A**) 12 weeks post-operatively immunofluorescence staining of isolectin B4 (IB4, yellow) for endothelial cells, microglia and macrophages, and Ki67 to visualize proliferating cells (upper panel, red). Arrows point to proliferating IB4-positive cell. (**B**) Laminin is used to show integrity of Bruch’s membrane (lower panel, red). Cell nuclei are stained with DAPI (cyan). INL, inner nuclear layer; ONL, outer nuclear layer. Scale bar = 100 µm

In the respective histology, the atrophy of the retina progressed in all 13 scraping sites up to the inner retinal layers. The choriocapillaris contained some scattered erythrocytes and the overlying Bruch’s membrane appeared contiguous on HE stainings (Fig. 7A and B). Microglia/macrophages lineages, which invaded the subretinal space in the first week after surgery, were not detectable at 12 weeks by IB4 (Fig. 8). In addition, the proliferation at the site of the RPE wound appeared to have ceased, as Ki67 signals were missing (Fig. 8). Vascular endothelial cells also stained by IB4 did not form vessel-like structures beyond the integer BM and the RPE layer (Fig. 8), confirming the observation made before in the OCT and angiography. Thus, this surgical manipulation did not lead to CNV formation, neither in the short-term nor in the long-term histological specimens (Figs. 5 and 8, respectively).

**Fig. 9:**
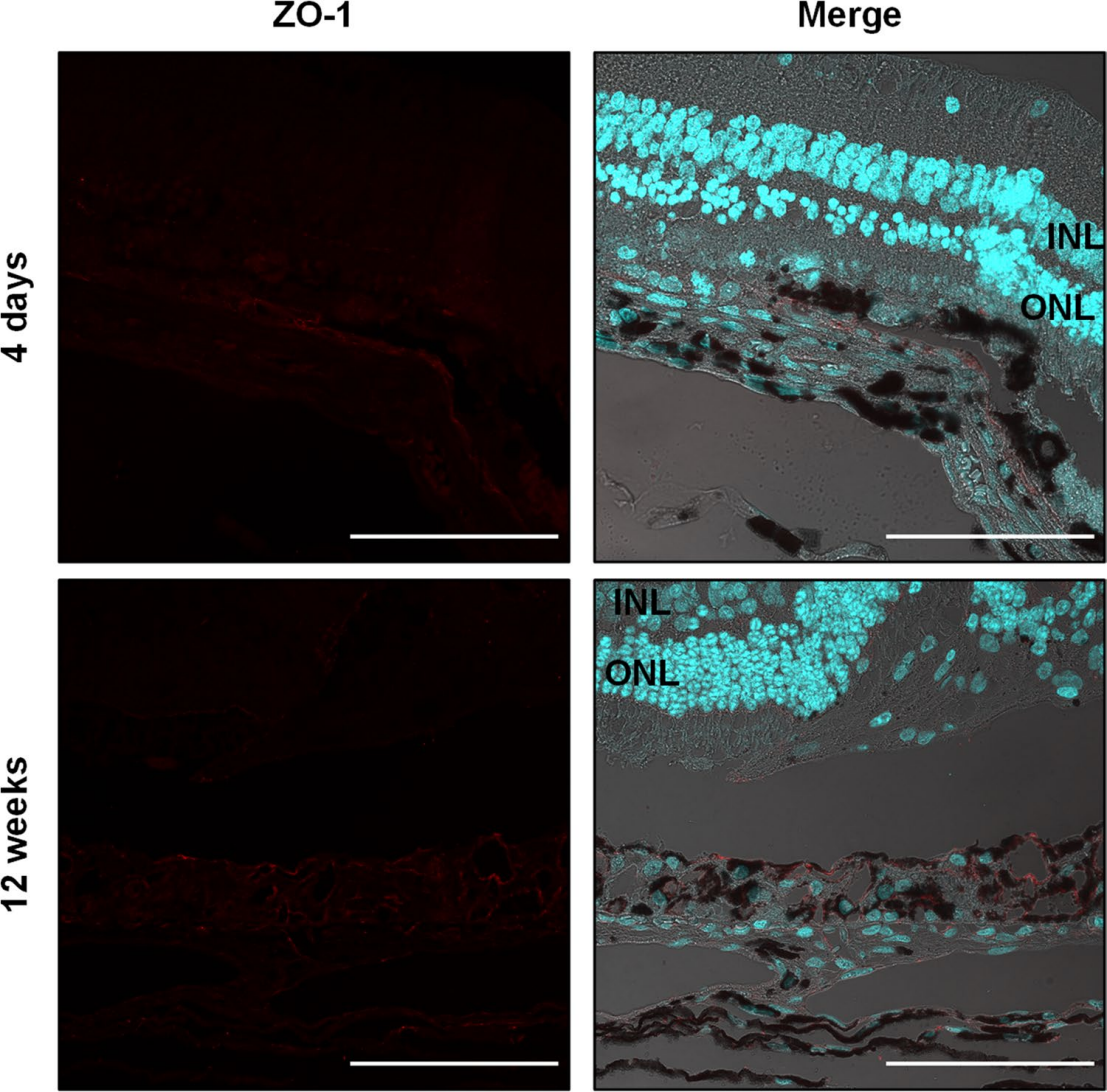
Immunofluorescence images of ZO-1 staining of retinas 4 days and 12 weeks after RPE debridement surgery. The images show the transition zone, which contains the site of the RPE wound and the neighboring untouched RPE. ZO-1 (red) is used as epithelial polarity marker and DAPI (cyan) to visualize nuclei. The merged image contains also the transmission light microscopic picture. INL, inner nuclear layer; ONL, outer nuclear layer. Scale bar = 100 µm

Interestingly, already 4 days after RPE removal, the wound has almost closed. However, the newly formed RPE cells beneath the retinotomy are hypertrophic. This appearance is unaltered after 12 weeks (Fig. 7C). We assessed the functionality of these cells by staining for ZO-1, which is an intracellular protein located at the apical tight junctions and is often used as a marker for correct epithelial polarity [19]. After 4 days, there is no ZO-1 staining at the site of the RPE wound, but in transition to the native RPE, it is expressed and mainly seen at the apical surface facing the photoreceptor layer (Fig. 9). After 12 weeks, the multi-layered RPE beneath the atrophic photoreceptor layer does express ZO-1, but there is no polarization visible. This phenotype extends also to the edge of the wound, where the ONL has a normal morphology (Fig. 9).

**Fig. 10:**
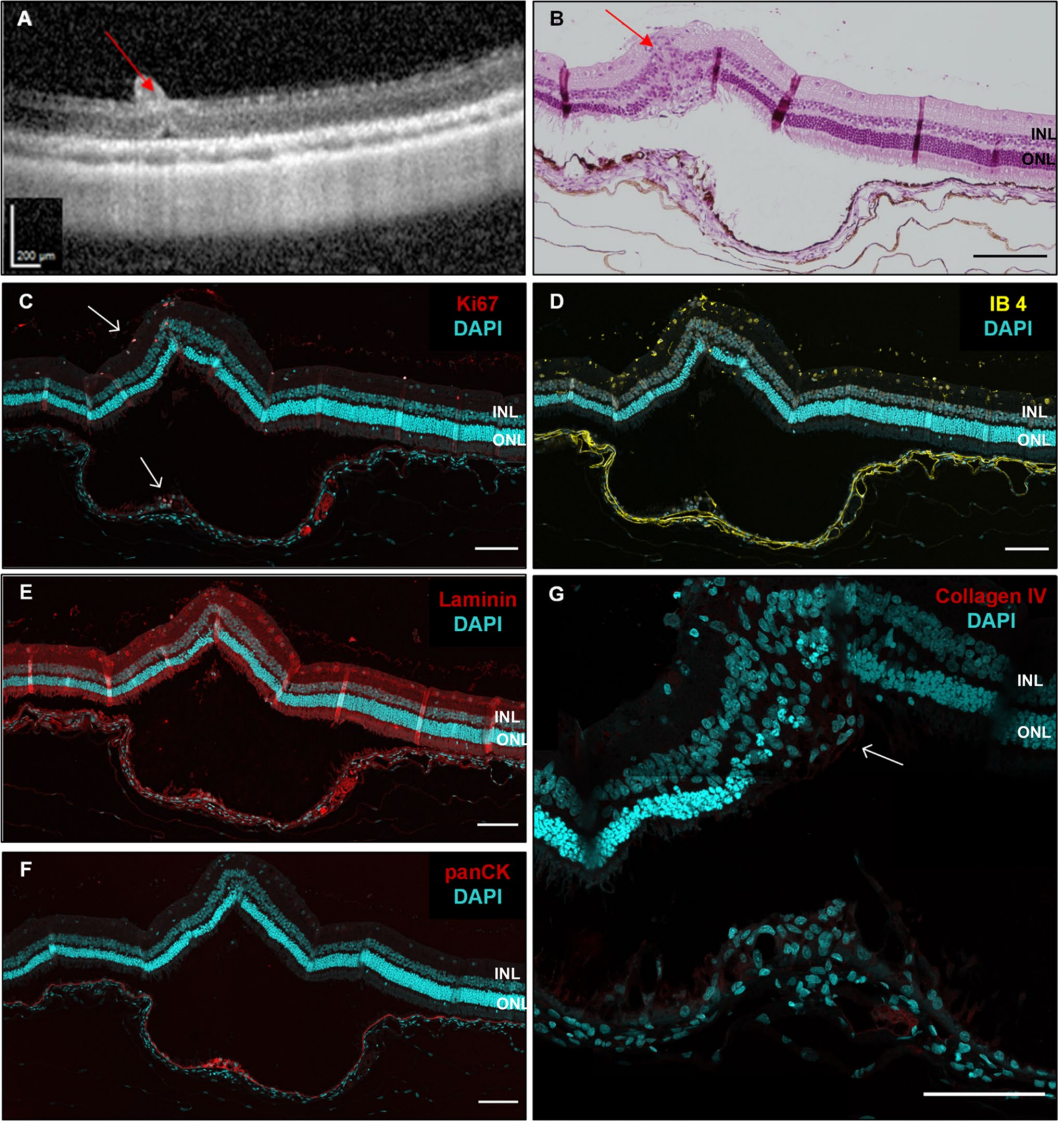
SD-OCT (**A**) and corresponding histology of a retina 12 weeks after generation of a bRD without scraping the RPE. HE staining, scale bar = 150 µm (B) and immunohistochemistry (**C**–**G**). Red arrows show subretinal injection site. White arrows indicate areas with ongoing cell proliferation (Ki67 staining, **C**). Without a generated RPE wound, there are only few infiltrating microglia/macrophages (IB4 staining, **D**). Laminin was used to visualize Bruch’s membrane (**E**) and panCK staining revealed a small spot of hypertrophic RPE beneath the bRD (**F**). Collagen IV is only found as small deposit in the outer nuclear layer (**G**); scale bar C-G = 100 µm

In our control group with a bRD without scraping after 12 weeks, a central pigment epithelial hypertrophy was observed only around the retinotomy (Fig. 10A, B, and F). We also saw cell proliferation in the RPE layer (Fig. 10C) that was not scraped during surgery, as well as few infiltrating microglia or macrophages in the outer nuclear layer and even more in the inner nuclear layer, where we also found ongoing cell proliferation (Fig. 10B and C). The collagen IV staining shows also only minor deposits in the ONL (Fig. 10 G). Confirming that the induction of a retinal detachment alone activates retinal wound healing processes [20].

## Discussion

We aimed to develop a localized outer retinal atrophy model induced by RPE removal to enable the translational preclinical animal studies of RPE cell therapeutics. While in our previous study on acute effects of surgical RPE removal, photoreceptor outer segments (POS) were intact [16], and we now saw atrophy of the ONL in all samples at 4 days and 3 months. This loss is attributed to the removal of the RPE layer since the generation of a bRD alone in our control did not lead to atrophy of the ONL. This was also reported in another study where long-term effects of bRDs alone in the rabbit retina were evaluated [21]. By contrast, Szurman et al. [22] reported that the immediate effects of a bRD are RPE monolayer disruption and torn POS. Similar results were also seen by another group [23]. Differences in surgical technique may explain the discrepancy between these outcomes, e.g., we utilize a 0.1–0.25 mL Hamilton syringe vs. 1 mL syringe, low intraocular pressure (IOP) in a valved and sutured port 25G system vs. 20G 3 port sclerotomy PPV, and complete removal of the cortical vitreous vs. just core vitrectomy. In the merangiotic retina of the rabbit, only a small part of the retinal nutritional supply comes from blood vessels, and photoreceptors are therefore dependent on RPE cells to supply nutrients and waste disposal (i.e., phagocytosis of POS), in addition to the other functions of the RPE cells in maintaining a healthy retinal environment [21]. With this observation, we met our first aim to generate a rabbit model for loss of photoreceptors due to a removed RPE.

To date, our group had the opportunity to perform similar techniques on 5 different rabbit strains, including chinchilla bastard, Chinchilla bastard/KBL hybrids, New Zealand White/Red Cross, New Zealand White (albino), and Dutch belted. Both male and female rabbits were operated on, while we did not register any gender specific differences in our results. For our experiments, we chose the Dutch belted strain having retinal pigmentation to allow contrast appreciation during subretinal implantation. It has also a thick sclera and larger eye volumes making the operation smoother. The histologic analysis revealed already 4 days after surgery a hypertrophic RPE cell layer that had grown back to close the surgically induced RPE wound. These RPE cells have an abnormal appearance, as we observed multi-layering and even after 12 weeks still a mounded appearance, which could be accompanied by at least a partial loss of function. A similar observance was made by Ivert et al. [21] after induction of a bRD without further manipulating the RPE layer. They demonstrated in their follow-up examinations the mounded appearance of the RPE cells after this surgery and that it induces breakdown of the blood-retinal-barrier, a crucial function of the RPE. The RPE is quite sensitive to changes in its microenvironment through so-called dynamic reciprocity reactions with the extracellular matrix [24], which might be the reason why the RPE cells, which closed the induced wound already after 4 days, have not yet regained their normal appearance after 12 weeks. During this wound-healing process, RPE cells might undergo epithelial-mesenchymal transition (EMT), where they lose their epithelial phenotype by rearrangement of their cytoskeleton, loss of cell–cell contacts, and polarity [25]. We attempted to asses polarity by ZO-1 staining and indeed did not observe a RPE monolayer with apical ZO-1 expression, as it would be in native RPE [19]. With our results, we cannot exclude that at least a partial EMT has occurred, as cytokeratins are conserved proteins of the RPE cytoskeleton [26], and the expression of the tight-junction protein ZO-1 was weak. For a clarification of these processes, in situ analysis of the transcriptional profile of the RPE would be needed.

Dying photoreceptors release damage-associated molecular patterns (DAMPs), such as HMGB1 [27], which activate innate immune cells and microglia. Arroyo et al. [28] showed that the peak of photoreceptor death in humans after retinal detachment was found around day 2, perhaps explaining why staining of our day 4 histologic samples for Caspase 3 yielded no signal. In steady state, microglia have a ramified appearance and reside in the plexiform layers where they scan the retinal environment. Upon activation, they change to an amoeboid form and migrate toward the relevant site [18]. Indeed, we could identify the presence of IB4-positive cells in the space between photoreceptors and choroid in the early post-operative phase colocalizing with strong collagen IV depositions (Fig. 5), but after 3 months, these cells were again mainly detectable in the IPL (Fig. 8, upper panel). It could be both microglia and macrophages, as both cell types are stained by IB4 and in general share common markers. It is most likely that the cells observed in the photoreceptor layer are microglia, as in a study of retinal detachment in cats macrophages were detected only in the subretinal space and did not infiltrate the retinal layers [20]. The advantage of the cat model in this case was that microglia in cats are negative for CD11b and, therefore, allow for the discrimination of these two cell types. Our experimental setup does not allow to determine the origin of the collagen IV depositions, given only 2 histologic time points were sampled. Wickham et al. observed also an invasion of microglia to the outer retina layers as well as an increasing number of GFAP-positive cells (astrocytes and Müller glia) with protrusions into the outer retinal layers after retinal detachment [29]. In another study, the same group also found ongoing proliferation activity in Müller cells and the activated microglia by BrdU labeling [30].

Technologies for RPE debridement have been described by other groups, such as hydraulic debridement [31]. This method showed promising results albeit with possible Bruch’s membrane breaks, yet no choroidal or inner retinal damage. The main setback of this method was the high rate of epiretinal membranes and proliferative vitreoretinopathy with rhegmatogenous retinal detachment in up to 20% of the cases in the 28 and 56 day groups [31]. Other groups attempting this method had further complications such as macroscopic [32] and microscopic [33] hemorrhages, fibrin, and cellular damage of the neural retina with intracellular edema [32]. Petrus-Reurer et al. [34] suggested a debridement with a subretinal injection of 50 µL sodium iodate causing RPE hyperautofluorescence, focal RPE loss, and choroidal atrophy and thereby mimicking the retina of a patient with geographic atrophy, yet even in the lowest dosage of 0.1 mM of sodium iodate caused a loss of the ellipsoid zone and outer limiting membrane bands as well as a thinning of the outer nuclear layer, even though the RPE cells were still intact [34]. Other studies also document the retinal toxicity of sodium iodate [35], thus banning its potential use in patients. In the former laboratory, outer retinal damage occurred also while subretinally injecting 50 µL BSS (= vehicle), confirming iatrogenic mechanical damage from their earlier report [23]. As discussed previously, we tried to overcome this problem by injecting a less amount of BSS (20–30 µL), as slow as possible, resulting in a flatter yet wide bRD, thereby reducing retinal stretching and photoreceptor damage (Fig. 9).

The rabbit model described here may serve as a reproducible and cost-efficient model for testing various stem cells derived therapies. Several studies have shown that rabbit models are suitable for evaluating human-derived stem cell therapy [36–38]. Even though functionality and survivability of human-derived RPE cells have been studied, one limitation of retinal atrophy models in rabbits is its merangiotic retina, which is distinguished by lack of retinal vessels as seen in Fig. 1a. This causes a faster and more severe atrophy than in animals with blood supply to the peripheral retina, yet this might be overcome by directly implanting RPE cells after scraping. Another limitation of the study is that the scraping was performed on healthy RPE without drusen or RPE-clumping and, therefore, may not be translatable into clinical trials due to safety concerns. Still, we were able to develop an improved large animal model for RPE debridement with minimal iatrogenic damage. This will form the basis for subsequent investigations with specific cell transplantations and carrier models. Future studies will investigate the survival und functionality of stem cell-derived RPE cells as a monolayer on a carrier or as suspension delivered to the RPE-wound and their effect on the above lying retina.

## Supplementary Information

Below is the link to the electronic supplementary material.

Supplementary file1 (TIF 536 KB)

Supplementary file2 (TIF 669 KB)

Supplementary file3 (TIF 970 KB)

Supplementary file4 (MPEG 15604 KB)

Supplementary file5 (M4V 13620 KB)

## Data Availability

Not applicable.
