# Supplementary figures and images for: A rabbit model for outer retinal atrophy caused by surgical RPE removal
Source: Graefes Arch Clin Exp Ophthalmol. 2023 Mar 28;261(8):2265–80. doi: 10.1007/s00417-023-06014-3 (PMC10368565; doi:10.1007/s00417-023-06014-3)

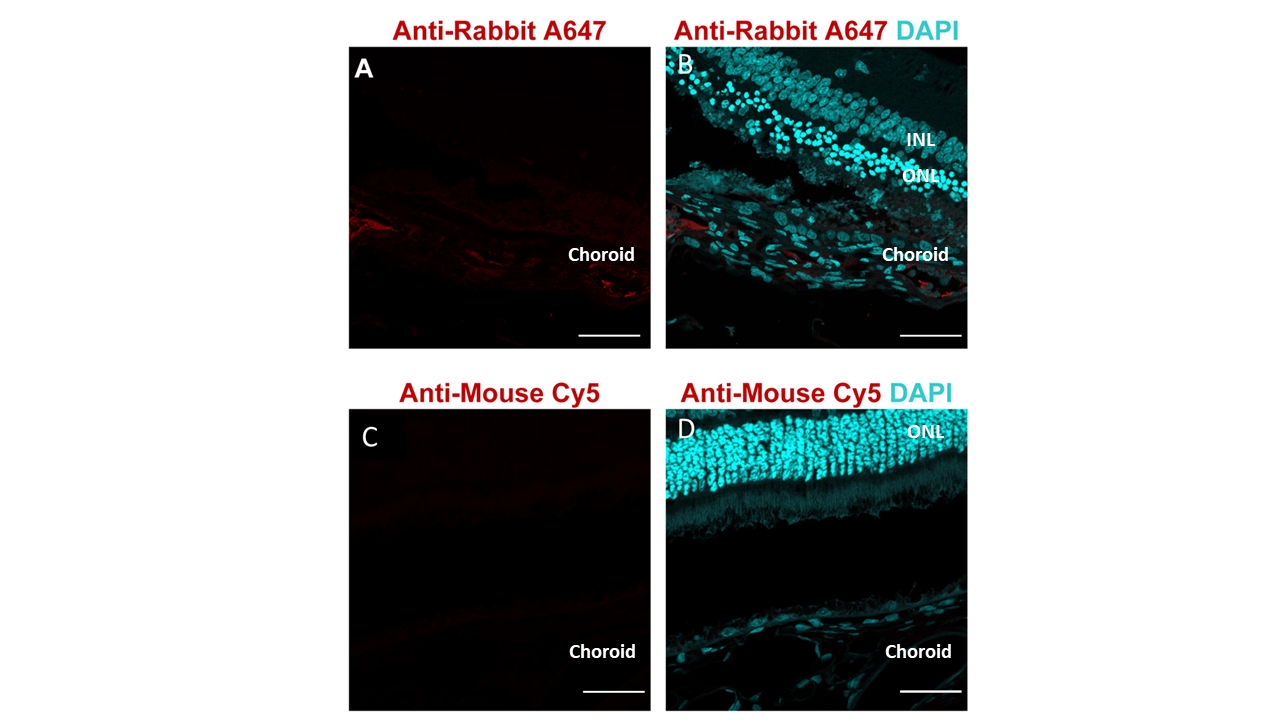

Supplement: Supplementary file 1 — Supplementary file1 (TIF 536 KB) [file 417_2023_6014_MOESM1_ESM.tif]

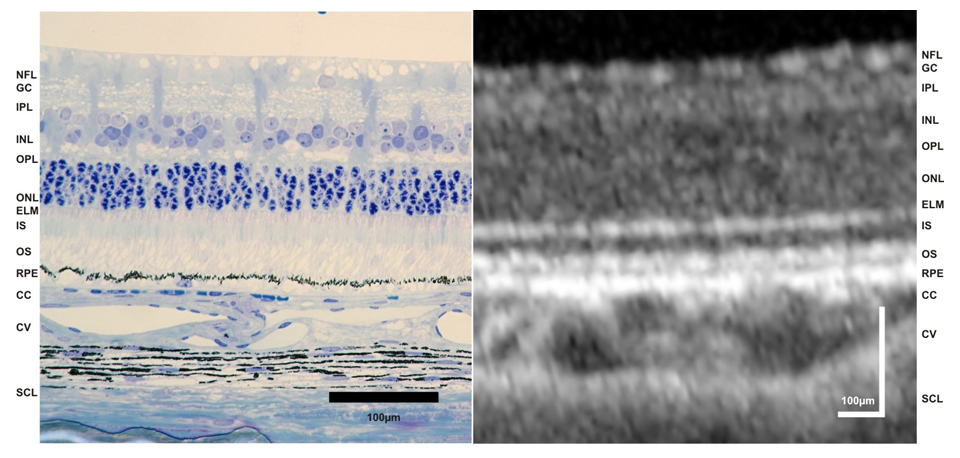

Supplement: Supplementary file 2 — Supplementary file2 (TIF 669 KB) [file 417_2023_6014_MOESM2_ESM.tif]

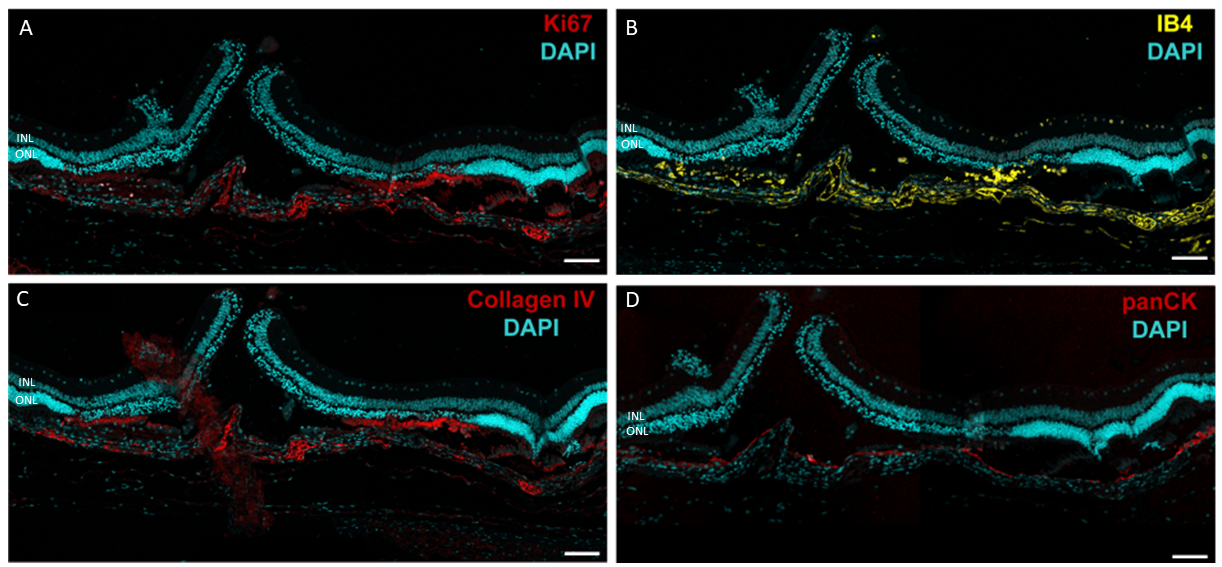

Supplement: Supplementary file 3 — Supplementary file3 (TIF 970 KB) [file 417_2023_6014_MOESM3_ESM.tif]
